# Use of low-protein staple foods in the dietary management of patients with stage 3–4 chronic kidney disease: a prospective case-crossover study

**DOI:** 10.1186/s12882-022-02734-6

**Published:** 2022-03-21

**Authors:** Junbao Shi, Yue Wang, Song Wang, Xinhong Lu, Xinxin Chen, Danxia Zheng

**Affiliations:** Department of Nephrology, Peking University Third Hospital, 49 North Garden Rd., Haidian District, Beijing, 100191 China

**Keywords:** Low-protein diet, Chronic kidney disease, Protein energy wasting

## Abstract

**Objective:**

Maintaining a low-protein diet (LPD) is important for patients with chronic kidney disease (CKD) to delay renal degradation and alleviate clinical symptoms. For most patients with CKD, it is difficult to maintain the necessary low level of dietary protein intake (DPI). To improve the current dietary management of CKD, we conducted an intervention study by administering low-protein staple foods (LPSF).

**Design and methods:**

We conducted a prospective case-crossover study among 25 patients with stage 3–4 CKD. During the initial 12 weeks of the study, we instructed the patients regarding a standard LPD according to the recommendations of a renal dietitian. In the second stage of the study, we requested the patients taking low-protein rice or low-protein flour (250 g/d) as an LPSF diet instead of regular staple food daily, and followed these patients up for 12 weeks. We compared the DPI, dietary energy intake (DEI), normalized protein equivalent of total nitrogen appearance (nPNA), serum creatinine levels, and nutritional index between baseline and the end of the study.

**Results:**

We found no change in dietary variables among the patients during the first 12 weeks of the LPD. After subjecting them to an LPSF diet, the corresponding variables showed a pronounced change. The patients’ DPI decreased from 0.88 ± 0.20 to 0.68 ± 0.14 g/kg/d (*P* < 0.01) and the nPNA value decreased from 0.99 ± 0.18 to 0.87 ± 0.19 g/kg/d (*P* < 0.01). The high biological value protein intake proportion increased from 42% (baseline) to 57% (*P* < 0.01) during the 24 weeks. No variation was found in the measured DEI (28.0 ± 5.8 vs 28.6 ± 5.4 kcal/kg/d), nutrition assessment, or renal function and serum creatinine levels.

**Conclusion:**

Our prospective case-crossover study demonstrated that an LPSF diet can help patients with stage 3–4 CKD reduce DPI and nPNA values, improve the proportion of highly bioavailable proteins, ensure adequate calorie intake, and avoid malnutrition. An LPSF diet is an effective and simple therapy for patients with stage 3–4 CKD.

## Introduction

Maintaining optimum protein intake to protect and enhance kidney function in patients with chronic kidney disease (CKD) has been a controversial topic for years [1–4]. High protein intake may aggravate the workload of the renal system and accumulate toxic protein metabolites, leading to a higher risk of kidney failure [3, 5, 6]. However, a very low-protein diet (0.28 g/kg/d) supplemented with a mixture of essential keto acids and amino acids is a double-edged sword, as it can delay renal deterioration by reducing metabolic burden but may conversely worsen renal deterioration or cause protein-energy wasting (PEW), which is associated with a high all-cause mortality rate [7].

A low-protein diet (LPD) is a better therapeutic alternative for controlling protein intake among patients with CKD. This dietary intervention offers a variety of clinical benefits, such as lowering serum toxins, ameliorating clinical symptoms, and delaying renal deterioration [5, 6, 8, 9]. For example, in an extended follow-up study, Modification of Diet in Renal Disease (MDRD), the most famous randomized controlled trial on the effects of an LPD in patients with CKD, the LPD group of patients with stage 3–4 CKD with a mean DPI of 0.77 ± 0.12 g/kg/d yielded fewer renal failure cases than the patients of the ordinary protein diet group (DPI 1.3 g/kg/d, hazard ratio 0.68) [10].

The commonly accepted standard range of DPI for patients with stage 3–4 CKD is 0.6–0.8 g/kg/d [5, 11–14]. However, for most patients with CKD, it is difficult to control their DPI as per the standard range, even under the guidance of registered dietitians. The MDRD study found that average DPI reached 0.77 g/kg/d under strict guidance, contrasting with the desired value of 0.58 g/kg/d in the LPD group [1].

A meal schedule of three meals a day, consisting of dietary staples, is indispensable in the treatment of CKD and undoubtedly plays a leading role in successful CKD treatment using the LPD. During the past two decades, low-protein staple foods (LPSF), as a relatively new method in the treatment of CKD, has attracted much attention [15, 16]. The results of a study by Mochizuki et al. indicated that patients with CKD are able to effectively adhere to an LPD without changing their dietary habits through the LPSF intervention [17]. LPSF is composed of ordinary rice or flour, processed by means of proteolysis to remove proteins while preserving the remaining unprocessed nutrients [16]. This may be a suitable intervention for patients who (1) have strictly implemented an LPD but cannot meet their calorie and protein requirements with ordinary staple foods, (2) have met their calorie and protein requirements through an LPD but cannot adhere to this diet for an extended time because of their dietary habits and lifestyle, or (3) have previously relied on starch staple foods to meet their calorie and protein requirements.

Unfortunately, only a few studies have explored the use of LPSF to effectively administer an LPD to patients with CKD, except for the aforementioned report from Japan [16, 17]. Therefore, we conducted a case-crossover study to reduce DPI among patients with stage 3–4 CKD through the LPSF diet.

## Methods

### Study design

We conducted a prospective case-crossover study, comparing the change of same patients receiving two different dietary interventions. The study involved two steps, (1) During the initial 12 weeks of the LPD study phase, we instructed the patients regarding a standard LPD according to the recommendations of a renal dietitian. (2) In the second 12 weeks of the LPSF study phase, we requested the patients taking low-protein rice or low-protein flour (250 g/d) as an LPSF diet instead of regular staple food daily without any other change to patients’ usual diet. Patients with stage 3–4 CKD were recruited from the outpatient department of the Peking University Third Hospital from June, 2017 to June,2018. The whole observation period lasted for 24 weeks. Eligible patients were volunteers aged 18–80 years with a stage 3–4 CKD diagnosis and an estimated glomerular filtration rate (eGFR) of 15-60 mL/min/1.73 m^2^. Exclusion criteria included a diagnosis of acute kidney injury, diabetic mellitus, or any other severe diseases (heart failure, active hepatic disease, digestive diseases with malabsorption, or inflammation); a blood pressure > 180/110 mmHg; and difficulty adhering to a therapeutic diet.

### Study protocol

**Fig. 1:**
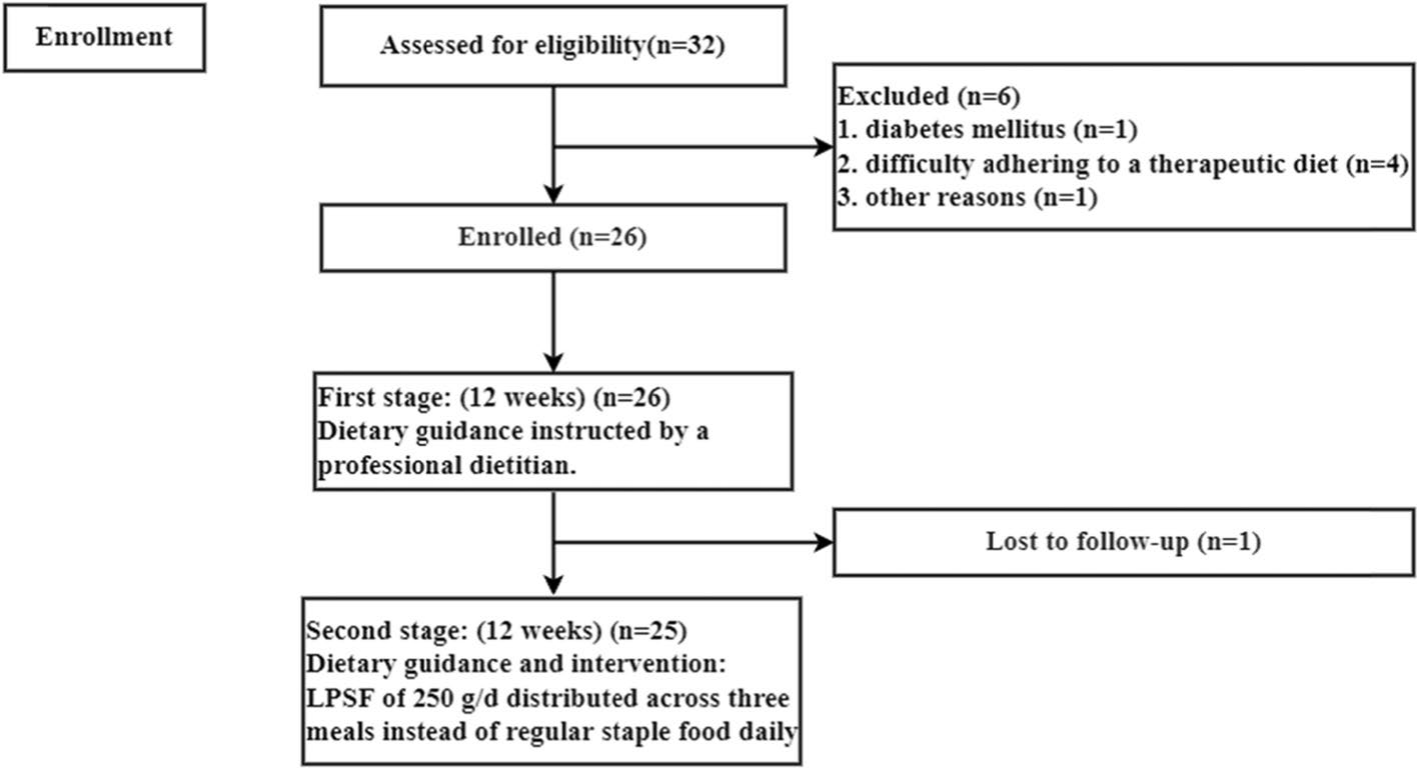
The flow chart of the participants in the study

All patients were directed to follow the LPD intervention with a DPI of 0.6–0.8 g/kg/d for 12 weeks, as instructed by a professional dietitian. Patients were administered a survey following this intervention to analyse their daily dietary intake and nutrition level. Patients were then requested to follow the LPSF intervention (Sinofn [Tianjin] Pharm-Tech Co., Ltd., Tianjin, China) for the following 12 weeks, with a LPSF of 250 g/d distributed across three meals, consisting of common staple foods. During this intervention period, all other aspects of their diet and medication regimen were continued as before the intervention study or adjusted by a medical care provider if needed. The flow chart of the participants in the study is shown in Fig. 1. The trial was approved by the Ethics Committee of the Peking University Third Hospital on 23 February 2017 and retrospectively registered at ChiCTR (ChiCTR2000030112) on 23/02/2020. The research protocol meets the requirements of the Helsinki Declaration.

### Sample size

The sample size required for this study was calculated using a paired t-test. The expected change in estimated DPI based on previous tests was 0.2 ± 0.3 g/kg/d, with α = 0.05 and β = 0.1. A total of 24 cases were required for the study to have sufficient statistical power. This power calculation was conducted using PASS 14 Power Analysis and Sample Size Software (NCSS, LLC., Kaysville, UT, USA).

### Food diary

All patients were requested to provide dietary records for 3 consecutive days every 4 weeks during the study, including at baseline, at 12 weeks after the first (LPD) arm of the dietary intervention, and at 24 weeks at the end of the study and LPSF intervention. To ensure the accuracy of the patients’ food diary records by themselves, all participants were received the extensive instructions from a renal dietitian. They were trained how to arrange three meals a day and record their dietary intake in detail. Moreover, a nurse was precipitated as an assistant who was responsible for reminding the patients about each step in the study, check-up the patients’ record, and help them to give the record correctly.. Dietary data, such as DPI, DEI, high biological value protein intake, and daily intake of phosphorus and potassium, were calculated using software based on the National Food Content Table (Information Management System, Peking University, Beijing, China). The DPI, DEI, and high biological value protein intake were normalized for the actual body weight of the patients. National Light Industry Food Quality Supervision and Inspection Tianjin Station conducted the analysis of the food ingredients, such as low-protein rice and low-protein flour. The LPSF provided in our study contained a low protein content of 0.4%; potassium and phosphorus levels were approximately 2.3 and 40% of that of ordinary rice, respectively. The ingredient list of low-protein staple food and common staple food is shown in Table 1.

**Table 1:** The ingredient list of low-protein staple food and common staple food

Nutrition Facts (per100g serving)	Low-protein rice	Low-protein flour	Common rice	Common flour
Calories (Kcal)	340	361	346	344
Protein (g)	0.4	0.4	7.4	11.2
Total Fat (g)	0.1	0.2	0.8	1.5
Carbohydrate (g)	79.3	88.9	77.2	71.5
Phosphorus (mg)	45	48	110	188
Potassium (mg)	2.4	20	103	190

### Laboratory data

Blood and urine samples were measured by Pecking University Third Hospital’s laboratory as routine laboratory test. The analyses measured clinical parameters including kidney function (serum urea, nitrogen, and creatinine levels), blood electrolytes (phosphorus, calcium, potassium, sodium, chlorine, and bicarbonate levels), haemoglobin, and serum lipid levels. Liver function tests, 24-h urine tests (protein, urea nitrogen, creatinine, phosphorus, and potassium levels) on diet-recording days, and routine urine tests were also performed. The nPNA was calculated as follows [18]: nPNA (g/d) = 6.25 × urine urea nitrogen (g/d) + 0.03 × weight (kg) + proteinuria. (g/d).

### Nutritional status assessment

The patients’ nutritional status was assessed by measuring the serum albumin level, body mass index (BMI), grip strength, upper arm circumference, triceps skinfold thickness, haemoglobin level, and adipose tissue index, and by administering the subjective global assessment (SGA). The SGA comprises six subjective assessments, three of which are based on the participant’s history of weight loss, anorexia, and vomiting; the remaining three, muscle wasting, the presence of oedema, and loss of subcutaneous fat, were accounted for in the physical examination. According to these assessments, the patients’ nutritional status was evaluated on a graded scale of 1–7 [14].

### Statistical analyses

All statistical analyses were conducted using SPSS software (SPSS 18.0, Chicago, IL, USA). Numerical variables within the descriptive analysis are reported as means ± standard deviations. Categorical variables are presented as absolute values. Statistical analyses were performed using the paired sample t-test. A *P*-value < 0.05 was considered statistically significant.

## Results

### Characteristics of the study participations

A total of 26 patients were enrolled in the study, one patient was lost to follow-up (1/26 = 3.8%) during the first 12 weeks of dietary instruction. Finally, 25 patients (15 males and 10 females) aged 28–78 years, with a mean age of 59 ± 14 years were included. Glomerulonephritis was the most common primary disease leading to renal failure among these patients. The aetiologies leading to renal failure among these patients were chronic nephritis (*n* = 11), hypertensive nephropathy (*n* = 7), chronic interstitial nephritis (*n* = 2), polycystic kidney disease (*n* = 1), kidney cancer surgery (*n* = 1), and unknown primary disease (*n* = 3). 88% (22/25) of patients were taking antihypertensive drugs and 54.5% (12/22) of them were taking angiotensin-converting enzyme inhibitors (ACEI) or angiotensin-receptor blockers (ARB), 24% (6/25) were receiving lipid-lowering medication. Descriptive statistics for these patients are summarized in Table 2. The changes observed for each indicator variable throughout the course of this intervention study are described below.

**Table 2:** Characteristics for 25 patients with LPD and LPD with LPSF intervention for 12 weeks each

Items	Baseline	LPD under dietitian’s instruction (12 weeks)	LPD with LPSF under dietitians instruction (12 weeks)
Nutrient intake			
DPI (g/kg/day)	0.88 ± 0.20	0.87 ± 0.19	0.68 ± 0.14^a*, b*^
DEI (kcal/kg/day)	28.0 ± 5.8	26.9 ± 5.0	28.6 ± 5.4
Phosphorus intake (mg/day)	917 ± 225	976 ± 351	805 ± 171^b**^
Potassium intake (mg/day)	1983 ± 697	1952 ± 636	1831 ± 675
nPNA (g/kg/day)	0.99 ± 0.18	0.97 ± 0.17	0.87 ± 0.19^a*, b*^
HBPI(g/kg/day)	0.37 ± 0.19	0.37 ± 0.12	0.39 ± 0.11
Proportion of HBPI	42%	43%	57%
Anthropometrics data			
Weight (kg)	66.8 ± 10.6	66.6 ± 10.4	66.7 ± 10.1
BMI (kg/m2)	24.4 ± 3.78	24.4 ± 3.76	24.5 ± 3.53
Waist-hip ratio	0.89 ± 0.06	0.88 ± 0.07	0.87 ± 0.07
arm circumference (cm)	27.5 ± 3.39	26.8 ± 2.56	27.3 ± 2.76
triceps skinfold (cm)	1.65 ± 0.85	1.51 ± 0.75	1.61 ± 0.85
arm muscle circumference (cm)	21.4 ± 5.15	22.1 ± 2.87	22.3 ± 2.36
Grip strength (left)(kg)	29.9 ± 7.22	30.1 ± 8.86	29.8 ± 8.35
Grip strength (right)(kg)	31.4 ± 7.00	30.8 ± 8.55	31.6 ± 8.53
Body muscle (kg)	47.1 ± 7.86	47.3 ± 8.18	46.9 ± 8.37
Body fat (kg)	17.1 ± 8.36	17.0 ± 8.36	17.1 ± 8.22
Laboratory data			
Serum creatinine (mmol/L)	165 ± 64.5	168 ± 71.8	171 ± 86
Serum phosphorus (mmol/L)	1.24 ± 0.19	1.24 ± 0.16	1.24 ± 0.18
Serum potassium (mmol/L)	4.52 ± 0.49	4.49 ± 0.45	4.48 ± 0.43
Serum albumin (g/L)	42.2 ± 3.46	41.4 ± 3.49	41.0 ± 3.88
TCHO (mmol/L)	4.46 ± 1.02	4.71 ± 0.86	4.68 ± 0.87
TG (mmol/L)	2.26 ± 1.58	2.45 ± 1.55	2.14 ± 0.96
HDL (mmol/L)	1.09 ± 0.33	1.12 ± 0.40	1.15 ± 0.34
LDL (mmol/L)	2.48 ± 0.83	2.68 ± 0.72	2.71 ± 0.51
eGFR (ml/min/1.73m^2^)	40.3 ± 13.8	41.5 ± 17.5	42.0 ± 18.0
Urine protein (g/24 h)	1.37 ± 1.64	1.56 ± 1.84	1.31 ± 1.40
Urine phosphorus (mmol/24 h)	19.6 ± 6.54	17.7 ± 6.48	18.2 ± 5.00
Urine potassium (mmol/24 h)	53.8 ± 21.4	48.4 ± 14.9	44.9 ± 19.0^a**^

^a^LPD with LPSF intervention Compared to baseline

^b^LPD with LPSF intervention Compared to LPD under a nutritionist’s instruction

*P < 0.01, **P < 0.05, (paired t-tests)

*HBPI* high bioavailable protein intake, *BMI* body mass index, *TCHO* total cholesterol, *TG* triglyceride, *HDL* high-density lipoprotein, *LDL* low-density lipoprotein, *eGFR* estimated glomerular filtration rate

### Dietary protein intake

**Fig. 2:**
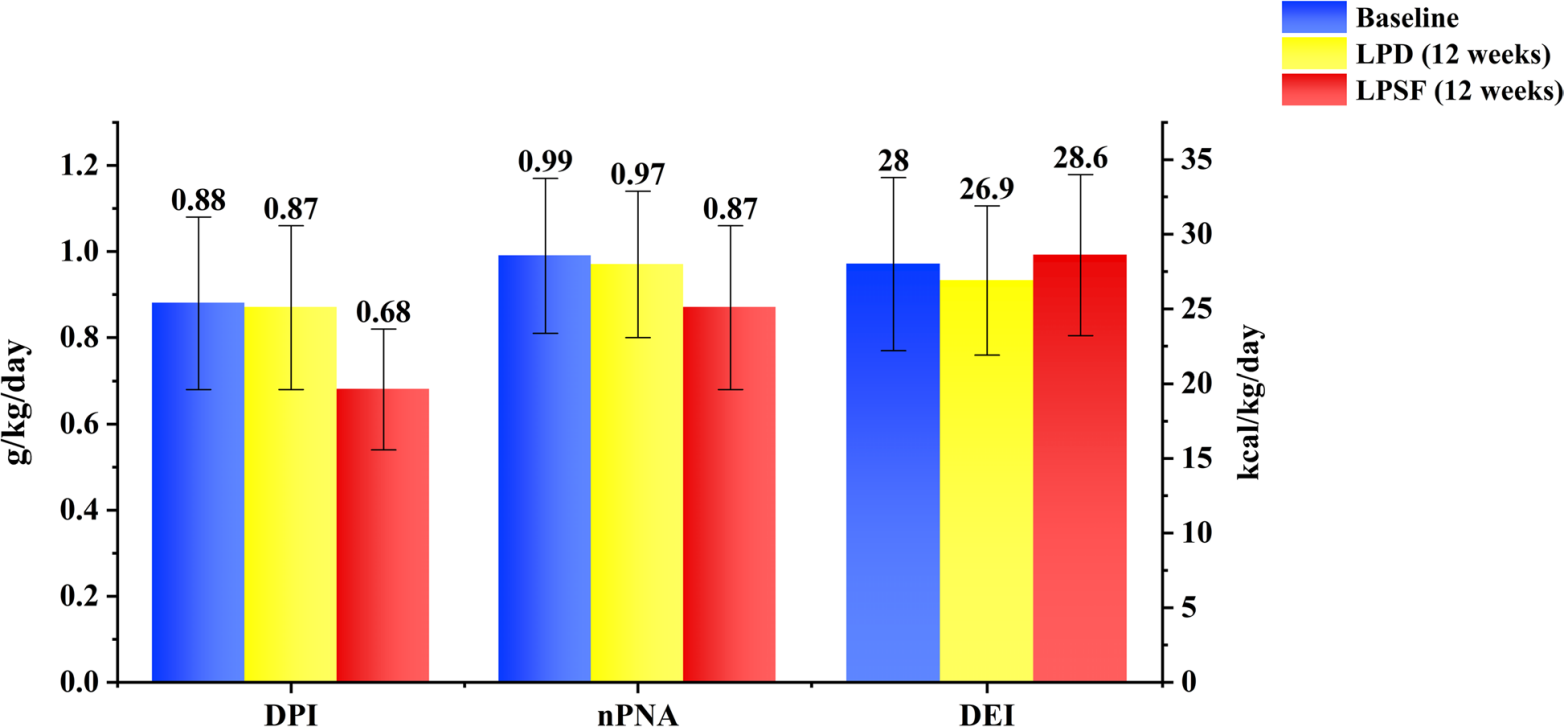
Comparisons of DPI, nPNA, and DEIvalues in different study phases

The patients’ mean baseline DPI was 0.88 ± 0.20 g/kg/d, which is slightly higher than the recommended value of 0.6–0.8 g/kg/d. This value did not change (*P* > 0.05) during the first 12 weeks of the LPD intervention. The following 12 weeks of the LPD with an LPSF intervention resulted in a significantly lower mean DPI (0.68 ± 0.14 g/kg/d) compared to the baseline data and the first 12 weeks of the LPD (*P* < 0.01) (Fig. 2). Although there was no difference in the intake of high biological value protein at 24 weeks, the proportion of high biological value protein intake of the patients increased from 42% at baseline (and the first 12 weeks) to 57% at 24 weeks.

### Normalized protein equivalent of total nitrogen appearance

The patients’ mean baseline nPNA was 0.99 ± 0.18 g/kg/d, and this showed no change (*P* > 0.05) after following an LPD for 12 weeks. After another 12 weeks of following an LPD with LPSF, the patients’ nPNA significantly decreased to 0.87 ± 0.19 g/kg/d (*P* < 0.01) (Fig. 2).

### Dietary energy intake

The DEI showed no change (*P* > 0.05) in the first 12-week LPD intervention period, as compared to the baseline mean DEI of 28.0 ± 5.80 kcal/kg/d. This DEI level was lower than the recommended value of > 30 kcal/kg/d, and continued to show no difference in the following 12 weeks of the LPD with LPSF intervention (mean DEI: 28.6 ± 5.44 kcal/kg/d at 24 weeks) compared to the DEI at baseline or at 12 weeks (Fig. 2).

### Renal function and serum phosphorus and potassium levels

**Fig. 3:**
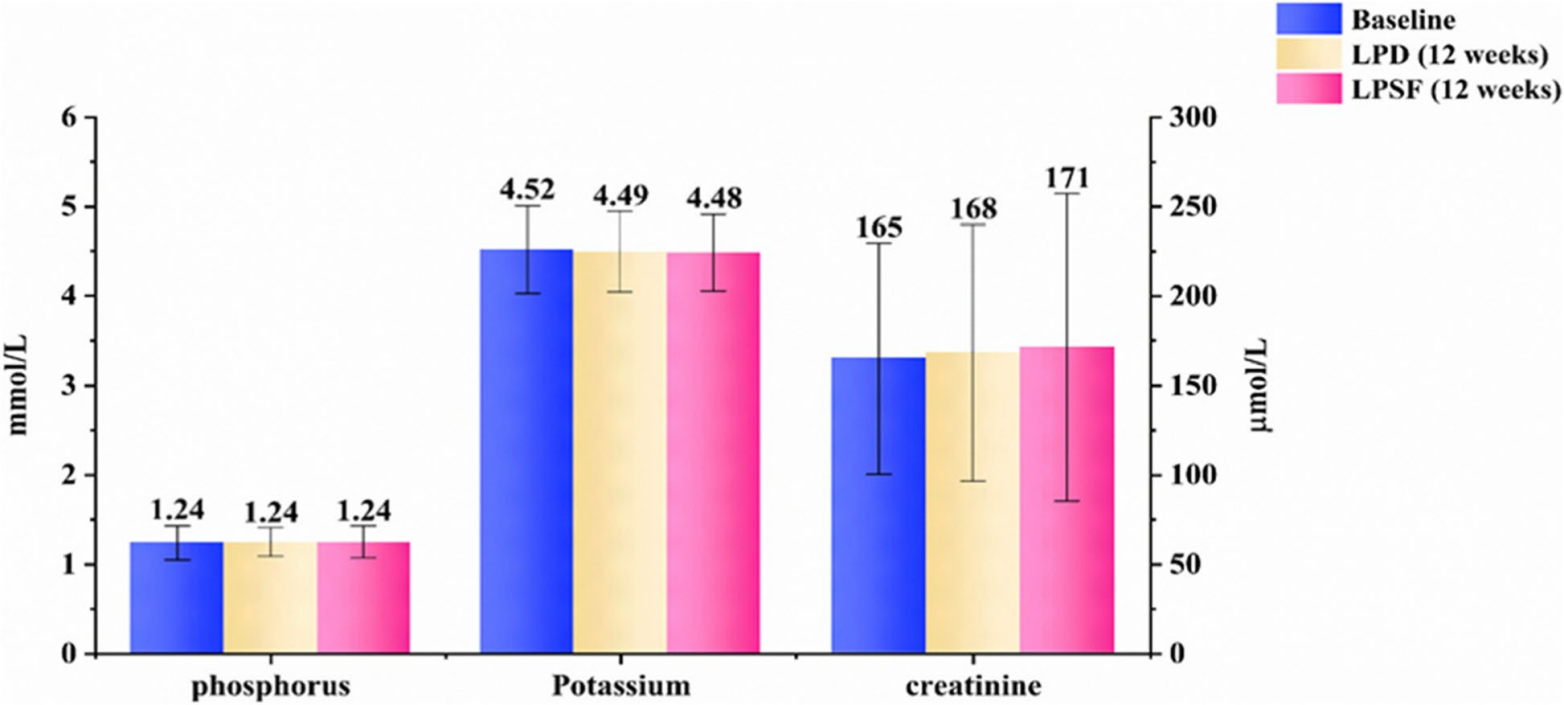
Comparisons of serum phosphorus, potassium, and creatinine levels in different study phase

We observed no significant change in the patients’ renal function throughout the study based on the eGFR or serum creatinine levels (Fig. 3). Furthermore, the serum phosphorus and potassium levels showed no change, despite the fact that patients had a substantially lower dietary phosphorus intake (805 ± 171 mg/d) during the LPSF intervention.

### Nutritional assessment

We conducted a nutritional assessment, which included measuring the BMI, serum albumin, waist-to-hip ratio, triceps skinfold thickness, grip strength, and SGA, none of which changed significantly during the observation period. Liver function and routine blood test results were unchanged. The use of prescription medication (data not displayed) continued as normal during the study.

## Discussion

A DPI of 0.6–0.8 g/kg/d, achieved when following an LPD with sufficient calorie intake (at least 30 kcal/kg/d), is optimum for patients with stage 3–4 CKD. This can improve azotaemia, reduce metabolic acidosis and related clinical symptoms, and may possibly delay the progress of CKD [5, 11–14]. However, the DPI is usually higher in patients with stage 3–4 CKD in the absence of dietary interventions. For example, the reported baseline DPI of patients with stage 3–4 CKD was 1.0 g/kg/d in the MDRD study [1]. Moreover, a large US sample survey with 16,830 respondents showed a DPI of 1.22 g/kg/d and 1.13 g/kg/d for patients with stage 3 and 4 CKD, respectively. These levels are much higher than the recommended standards and pose a substantial clinical and mortality risk [13].

There are several challenges to consider for a successful LPD as a CKD intervention. One important factor is the recommendation and medical judgment of the nephrologist with regard to an LPD. Some physicians do not actively recommend an LPD because it not only requires the additional support of clinical renal dietitians but may also cause malnutrition when combined with the many possible complications of CKD. Another factor depends on the patient’s persistence in adhering to an LPD. An LPD changes the eating habits of patients with CKD and requires long-term maintenance. In addition, the necessity of calculating calories for each food item accurately within the LPD intervention may confuse patients and lead to suboptimal dietary adherence [13, 19].

In our study, the patients’ mean baseline DPI was 0.88 ± 0.20 g/kg/d, reflecting the standard situation of patients with stage 3–4 CKD in the absence of intervention. The mean DPI showed no obvious change during the first 12 weeks of following an LPD, similar to the results of the MDRD study [3]. The following reasons may account for these results. (1) For most patients, no current standard method can efficiently maintain adherence to an LPD. Traditional methods, though being effective and low cost, are difficult for patients to tolerate for a prolonged duration. (2) It is challenging for most patients to change their dietary habits. (3) The amount of attention required to maintain an LPD effectively under the direction of a professional dietitian is challenging for most patients. Low compliance to an LPD is a subjective factor that more often than not continues to occur following objective efforts.

In order to solve these difficulties, we focused on the LPSF intervention evaluated here for managing patients’ DPI, which is a simpler, more patient-friendly method for reaching the LPD target [15, 16]. For a patient who weighs 60 kg, the daily protein intake of 250 g staple food in the LPSF intervention should be 0.3 g/kg less compared with normal protein staple food, if absorption is 100%. According to the research plan, the patients were requested to consume LPSF while retaining all other foods included in the 12-week LPD intervention. We expected that the patients would have a DPI decrease of 0.2–0.3 g/kg/d by consuming LPSF instead of regular staple food daily.

As expected, in our study after consuming LPSF for 12 weeks, both the mean DPI and nPNA of these patients significantly reduced as compared to before. Meanwhile the proportion of DPI below 0.8 g/kg/d increased from 48 to 84%, though DPI and nPNA reduced after 12 weeks of consuming LPSF, which strongly supports that LPD compliance improved with the inclusion of LPSF. In a Japanese study on low-protein rice consumed mainly as the staple food, 9 patients with CKD showed not only a decrease in their protein intake, but also a reduction in the reciprocal serum creatinine slope during the study period (mean of 7 months) [17]. In the current study, no change in kidney function was observed, perhaps due to our short observation time.

It is known that insufficient DEI may trigger PEW in patients with CKD, especially among those with inflammation, acidosis, and endocrine disorders. Differing from traditional malnutrition, the diagnostic criteria of PEW include low blood biochemical indicators (albumin, prealbumin, and total cholesterol), changes in body composition (reduced BMI, body fat percentage, and weight), decreased muscle mass, and insufficient dietary intake (low DPI and insufficient calories) [20, 21]. The development of PEW depends on a number of factors. Residual renal function decreases concurrently with DPI and DEI, which may cause more complicated diseases and induce PEW. In the MDRD study involving 1785 patients with middle and advanced stage CKD, the researchers found that their DPI, energy intake, and general nutritional status decreased simultaneously with a decrease in GFR [22]. Another study found that patients with stage 3–4 CKD had an increased risk of low levels of serum albumin and muscle mass accompanied by a low DEI after following an LPD (0.7 g/kg/d) for 6 weeks [21]. In addition, with sufficient calorie intake, the nutritional indices in the group with a mean DPI of 0.55 g/kg/d were similar to that in the group with a mean DPI of 0.8 g/kg/d, with no PEW occurrence [23].

Regarding this evidence, we paid special attention to calorie intake in the patients following an LPD. An important objective of the LPSF intervention in our study was for patients to achieve a 50% higher biological value for protein intake (mainly from animal proteins). To this end, the patients were encouraged to consume high-quality protein sources, such as milk, eggs, and meat, to increase their total calorie intake. After the patients switched to the LPSF intervention in our study, their average DPI decreased although their DEI did not show a clear reduction (28.6 ± 5.44 vs 26.9 ± 5.01, *P* > 0.05). The proportion of high biological value protein increased owing to the decrease in low biological value protein consumption from staple food. It can thus be concluded that the LPSF intervention more easily achieves a lower DPI, with an unchanged DEI and high biological value protein intake in patients with stage 3–4 CKD. The PEW indicators, such as serum albumin, total cholesterol, BMI, body fat, weight, and muscle volume showed no significant change (Table 2), indicating that the LPSF diet had no influence on PEW.

Potassium and phosphorus levels in LPSF are approximately 2.3 and 40% of those of ordinary rice, respectively. Given the low amount of phosphorus and potassium in staple food, even within a standard diet, the impact on blood phosphorus and potassium levels can be ignored even if these levels are significantly reduced.

We have also solved some issues regarding the administration of the LPSF diet, including (1) providing single servings of packaged rice from the cooperating factory to simplify cooking for the LPSF intervention, (2) developing low-protein dumpling flour and noodles to enrich LPSF varieties, and (3) allowing some patients to combine LPSF with different ratios of ordinary staple food in the study to give them comfort. It is difficult for patients to maintain the LPSF intervention at each meal, and ordinary staple food alternated with LPSF is a practical and optimal option.

This study has some limitations. First, the short duration of the trial did not permit conclusions on the long-term safety of LPSF therapy. It would take several years of observation to conclude that LPSF therapy can avoid malnutrition. Second,selection biases were unavoidable in this case-crossover study. Therefore, randomized controlled double-blind trials are more conducive to avoiding the bias of the research subjects.

In summary, we successfully administered an LPSF dietary intervention as a therapeutic method to manage the LPD for patients with stage 3–4 CKD. This intervention reduced patients’ intake of staple food protein, improved their proportion of highly bioavailable proteins, ensured sufficient energy intake, and prevented the occurrence of PEW. Furthermore, LPSF was shown to be beneficial in maintaining dietary habits and improving LPD compliance in patients with CKD.

### Practical application

A low-protein diet was recommended for stage 3–4 chronic kidney disease management in the present study. Low-protein staple food therapy can help patients with chronic kidney disease effectively control protein intake, but it may not be beneficial for patients who consume limited staple foods in their diet.

## Data Availability

The original data used and/or analyzed during the study are available from the corresponding author on reasonable request.
